# Social calls of the little auk (*Alle alle*) reflect body size and possibly partnership, but not sex

**DOI:** 10.1098/rsos.230845

**Published:** 2023-09-20

**Authors:** Anna N. Osiecka, Elodie F. Briefer, Dorota Kidawa, Katarzyna Wojczulanis-Jakubas

**Affiliations:** ^1^ Department of Vertebrate Ecology and Zoology, Faculty of Biology, University of Gdańsk, 80-308 Gdańsk, Poland; ^2^ Behavioural Ecology Group, Section for Ecology and Evolution, Department of Biology, University of Copenhagen, 2100 Copenhagen, Denmark

**Keywords:** information coding, partner similarity, seabird, source–filter theory, vocal communication

## Abstract

Source–filter theory posits that an individual's size and vocal tract length are reflected in the parameters of their calls. In species that mate assortatively, this could result in vocal similarity. In the context of mate selection, this would mean that animals could listen in to find a partner that sounds—and therefore is—similar to them. We investigated the social calls of the little auk (*Alle alle*), a highly vocal seabird mating assortatively, using vocalizations produced inside 15 nests by known individuals. Source- and filter-related acoustic parameters were used in linear mixed models testing the possible impact of body size. A principal component analysis followed by a permuted discriminant function analysis tested the effect of sex. Additionally, randomization procedures tested whether partners are more vocally similar than random birds. There was a significant effect of size on the mean fundamental frequency of a simple call, but not on parameters of a multisyllable call with apparent formants. Neither sex nor partnership influenced the calls—there was, however, a tendency to match certain parameters between partners. This indicates that vocal cues are at best weak indicators of size, and other factors likely play a role in mate selection.

## 1. Introduction

Finding a mate in a crowded colony can be a challenge. Acoustic signals can travel long distances and often provide cues to the caller's sex [1–3] and size [4,5], and are thus a great candidate for facilitating mate selection in dense, populous groups. The source–filter theory of vocal production postulates that sounds generated at the source (larynx or syrinx) are subsequently resonated by the filter (vocal tract), shaping the output spectrum of the call [6]. Depending on the length of the vocal tract, specific frequencies are dampened or enhanced, creating a stronger (amplified) output signal at certain frequencies, i.e. formants (resonances of the vocal tract), while others are filtered out [6]. While vocal tract elongations are used in some species to falsely indicate a larger body size [7], in general, both source- and particularly filter-related sound parameters are good indicators of body size, and are negatively correlated to it [8–10]. Although the source–filter theory was originally proposed for mammals [6], the importance of formants has been demonstrated in some bird species [10–14], including indication of size [10] and identity [10].

Body size information in vocalizations could be used in some species to achieve assortative mating, which consists in matching of certain, e.g. morphological [15–18] or physiological [17], traits between partners. In some cases, assortative mating is known to lead to certain advantages, such as improved offspring condition [19] or reproductive success [20,21]. While assortative mating tends to be somewhat overestimated [22], it is not very common in birds, compared to other taxa [23]. Nevertheless, it occurs across different seabird groups: species such as the long-tailed jaeger (*Sterocorarius longicaudus*) [24], Scopoli's shearwater (*Calonectris diomedea*) [18], Magellanic penguin (*Spheniscus magellanicus*) [25], the masked booby (*Sula dactylara*) [16] or the little auk (*Alle alle*) [17], all select their nesting partners according to certain morphological similarities, ranging from wing length [17], to foot colour [16]. Therefore, if the vocalizations of an assortatively mating species reflect traits such as body size, it can be expected that partners will also be similar vocally.

Vocal behaviour in birds can be influenced by hormones [26], and is often sex-specific. Also call parameters can—but do not necessarily have to—depend on sex. Across species, this information can be coded differently [1], such as using temporal [2,3] or spectral [1,27] parameters. Where a significant sexual dimorphism is present, vocalizations are also likely to differ—however, it can also assist in locating a potential mate in species with no sexual dimorphism.

**Figure 1.**
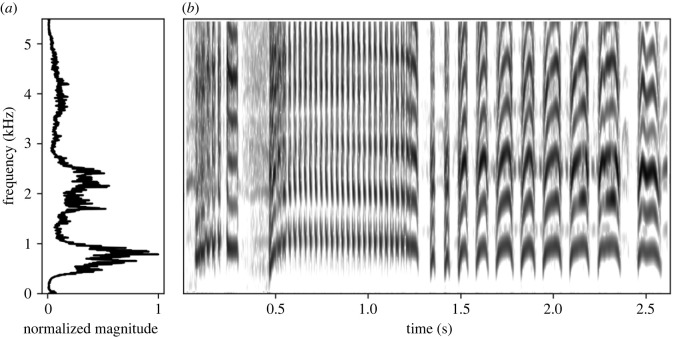
Spectrogram (*b*) and energy content at different frequencies (*a*) of a sample *classic call*.

Little auks are long-lived seabirds, nesting in densely populated colonies counting up to hundreds of thousands of individuals [28]. While they choose mates that are morphologically or physiologically similar to themselves [17], and usually maintain partnership over many years [29,30], nothing is known as to how these bonds are formed or are maintained over time, e.g. how potential mates are identified considering the lack of external dimorphism [30]. Little auks are very vocal, and use a variety of call types that vary significantly in their acoustic properties [31]. Most of these calls have a harmonic structure, and in the case of the *classic call* we can observe formants [31] (figure 1). Additionally, little auk calls change throughout ontogeny, with spectral parameters reflecting growth in chicks [32]. Vocal cues are thus a good candidate for coding socially important information, such as size and sex, in this species.

**Figure 2.**
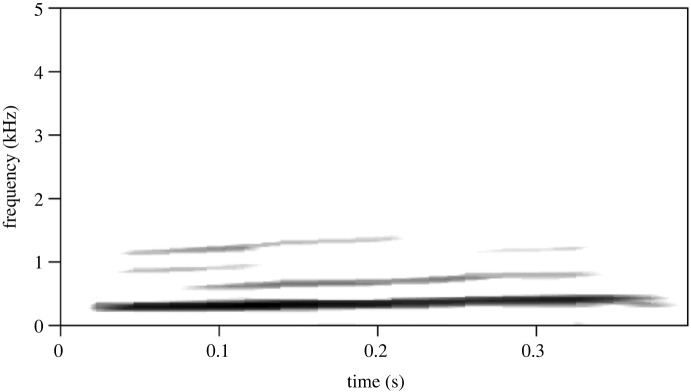
Spectrogram of a *short call*.

In this study, we investigated the information encoded in the source- (fundamental frequency, i.e. the lowest frequency of the sound, hereafter *f*0) and filter-related parameters (formants) of the little auk social calls. We selected two commonly used social call types: the *short call* (a simple, one-syllable call with no formants; figure 2) and the *classic call* (a complex, multi-syllable call with clear formants; figure 1), both used in a range of social interactions [31]. While *short calls* are used in close-range communication in or near the nest, the *classic call* is likely a long-distance call, often uttered by birds in flight but also used from inside or outside the nest [31]. Because of the frequency of their use, we selected them as socially important calls. Their very different spectral structures, on the other hand, suggests that these calls might carry different types of information. We examined whether source- (both call types) and filter-related (*classic call*) parameters could be cues to size, and whether partners' vocalizations are more similar than those of random birds. We have also tested whether sex affected the acoustic parameters of social calls.

## 2. Methods

### 2.1. Choice of the size proxy

Body size is usually measured based on the individual weight, selected skeletal proxy, or a set of measures [5,7,8,33,34]. Since the little auk's weight fluctuates heavily throughout the breeding season [35], it is not a good indicator of the overall size. Instead, we decided to use a single stable measure—the total head and beak length (THL). It is a good proxy of size in the species [36], and correlated between partners [36] (but see [17]). Moreover, containing a part of the vocal apparatus, it has the added advantage of being related to the vocal tract length.

### 2.2. Study site and recording set-up

All data were collected in the little auk breeding colony in Hornsund, Spitsbergen (77°00′ N, 15°33′ E), over two consecutive breeding seasons (2019–2020). All birds (two per nest, 18 nests in total) were handled (ringed with a unique combination of colour rings and measured) at the beginning of each field season. THL was measured using standard callipers as the distance between the back of the skull and the tip of the beak, with a 1 mm precision. The same person measured all the birds in the two seasons. If the captured individual was not yet known (i.e. had not been ringed before), aside from ringing and taking measures, its feathers were collected for molecular sexing, following a protocol adjusted to feather samples [36].

Recording little auk vocalization imposes a challenge as individual birds do not vocalize that frequently, and rather unpredictably in space and time. Moreover, vocalizing birds are often surrounded by other vocalizing individuals, creating unwanted noise in their recordings. Thus, for the purpose of this study, recording sessions were performed passively and in a continuous manner during the incubation period, with microphones inserted into the nest chamber. This way all the vocalizations produced inside the nests by focal adults (i.e. of known identity) were collected. Each nest was monitored during three different stages of incubation (early, mid and late). All sessions lasted 48 h, aiming to space them equally in time (i.e. about 8 days in between sessions) for all the monitored nests.

Audio recordings were made with an Olympus ME-51S stereo microphone (frequency response 100–15 000 Hz) placed inside the nests in such a way as to not disturb the birds. The microphones were connected to Olympus LS-3 or LS-P4 digital voice recorders (sampling rate 48 kHz, 16 bits) placed outside the nest and hidden under rocks. Synchronized video material was collected using cameras (commercial HD model of JVC, Japan; time-lapse mode: 1 frame s^−1^) placed in front of the entrance to each nest, to control for the identity of the focal individuals.

### 2.3. Data selection

Video recordings were reviewed in VLC software, noting the exact time each marked individual entered or left the nest. Since the birds were equipped with a unique pattern of colour-mark rings in addition to the standard numbered rings, it was possible to know which individual exactly was observed.

Then, the time intervals at which only one individual was present inside the nest were established using a custom-made script, and used to extract the corresponding audio fragments. This audio material was then manually reviewed in Raven Pro 1.6.4 (Cornell Lab of Ornithology, Ithaca, NY, USA), extracting all individual vocalizations recorded inside the nest. Great care was taken to not accidentally include vocalizations coming from outside the nest (i.e. of lower amplitude and/or audible sound distortion due to the burrow's walls), or vocalizations masked by noise. The resulting extracted vocalizations could therefore be assigned to individual of known sex, size, and breeding partner. We managed to obtain calls from 15 out of the 18 monitored nests, and both partners were successfully recorded in 11 nests (electronic supplementary material, table S1). Because we relied on the spontaneous vocal production of wild animals in a challenging recording set-up, the final sample sizes vary between call types and individuals, ranging from 1 to 70 calls extracted per individual (electronic supplementary material, table S1).

Little auks produce eight different call types [31], whose functionality is not yet well understood [31]. For this study, we selected two common social call types of a very different structure and contexts of use, i.e. the *short call* used in close-range social communication, and *classic call*, likely a long-distance call, used over a wide spectrum of contexts. This choice was made to include common calls that likely convey different types of information.

### 2.4. Sound analysis

To extract a standard set of 16 acoustic parameters (electronic supplementary material, table S2), all calls were analysed in Praat software [37], using a script [38–40] adjusted to the little auk [31] (electronic supplementary material, text S1), with the following spectrogram settings: Hann window, FFT-length = 715.

Additionally, mean values of the four first putative formants (F1–F4) were extracted from the *classic* calls using the FastTrack plug-in [41] for Praat, using the following settings: lowest analysis frequency = 500 Hz, highest analysis frequency = 7550, number of steps = 20, number of coefficients for formant prediction = 5, number of formants = 4. The formant dispersion, i.e. the averaged difference between successive formant frequencies, was then calculated as Fd=((F2−F1)+(F3−F2)+(F4−F3))/3*.* The number of extracted formants was decided based on visual assessment of the calls' spectrograms as well as script efficiency (i.e. more than four formants were never extracted by the script, and the extracted values were most reliable with those settings).

### 2.5. Statistical analysis

All analyses were performed in R environment (v. 4.1.3 [42]). The full data used in this study can be found in the electronic supplementary material.

### 2.6. Size

We used linear mixed models (LMMs; *lmer* function in *lme4* package [43]) to investigate the possible effect of size on the source- and filter-related acoustic parameters. These models included THL and sex as fixed factors (where sex was used as a control factor), and ID as a random factor to control for repeated measures. To avoid running multiple models on each parameter separately and hence avoid risks of type I error, we chose to test the effect of body size on one representative source-related parameter, the mean *f*0 value across the call (hereafter *mean f*0), and one representative filter-related parameter, the formant dispersion. Those parameters were chosen since they are usually reliable indicators of body size across taxa [44]. We prepared two models for the source parameter: one for the *short* and one for the *classic* call type. For the filter parameter (formant dispersion), we prepared one model (only *classic* call type). In the LMMs, we used the *PBmodcomp* function (*pbkrtest* package [45]), comparing models with and without THL included, i.e. providing *p*-values for the compared parameter.

### 2.7. Sex

First, we performed a Kaiser–Meyer–Olkin test on raw parameters of the *short* and *classic* calls separately (function *KMO*, *psych* package [46]). Since the overall MSA was higher than 0.5 [47] for both call types (MSA_short call_ = 0.75; MSA_classic_ *=* 0.57; electronic supplementary material, table S3), a principal component analysis (PCA) was performed (function *prcomp, stats* package [48]) on the 16 extracted acoustic parameters (electronic supplementary material, table S2) to reduce data dimensions. The scores of the PCA with eigenvalues >1 (Kaiser's criterion) were then used as input data for the following tests (the first five PCs for the *short call,* and first six PCs for the *classic call;* electronic supplementary material, table S4).

To investigate the influence of sex on the acoustic parameters of the calls, we analysed the data using permuted discriminant function analysis (pDFA). The dataset was based on multiple sampling per individual. The use of a pDFA allowed us to test the effect of sex (test factor) on the PC scores (input variable) while controlling for repeated measures of the same individuals (included as a control factor). A pDFA with nested design was conducted using the *pDFA.nested* function (R. Mundry, based on function *lda* of the MASS package [49]). The pDFA randomly selected calls for each combination of test and control factors. This random selection was repeated 100 times, and results were averaged. The number of permutations was set at 1000 (default). This procedure was run separately for the *short* and *classic* call types.

Because temporal information can be very important in coding cues to sex in seabirds [2,3] but sound duration did not strongly contribute to the PC scores used in the pDFA (electronic supplementary material, table S5), we additionally used LMMs (*lme4* package [43], *lmer* function) including sound duration as a response variable, sex and THL as fixed factors (where THL was used as a control factor), and ID as a random factor to control for repeated measures. To obtain *p*-values of the LMMs, we used the *Pbmodcomp* function (*pbkrtest* package [45]), comparing models with and without sex included. This was done separately for the *short* and *classic* call types.

### 2.8. Partner similarity

We used a correlation analysis to compare vocal similarity between nesting partners versus randomly assigned individuals. For this, we used the mean *f*0 values and sound duration of *short* and *classic* call types, formant dispersion in the *classic* calls, as well as the scores of the first PC of each call type.

First, all parameters were averaged for each individual. Average values of partners were then compared using Spearman's correlation test (observed values; *cor* function in *stats* package [48]). To establish significance of the observed values, a randomization procedure was performed separately for each parameter, where males and females were shuffled to create random pairs. For those, correlation coefficient was calculated (randomized values; *cor* function, *stats* package [48]); the procedure was repeated 1000 times. The *p*-value was calculated as the proportion of randomized values that generated a correlation equal to or more extreme (in absolute terms, i.e. values equal or higher for positive correlations) than the correlation obtained from original male–female pairings: p=1−(sum(observed values≥randomized values)/N). Because of the multiple testing, we used Bonferroni adjustments, so that *p*-values retained significance at 0.007 (i.e. 0.05/7).

## 3. Results

### 3.1. Size

**Figure 3.**
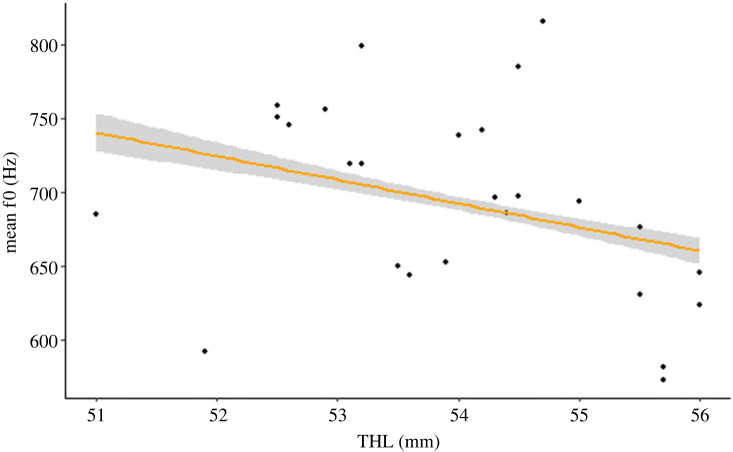
Relationship between size (total head length) and mean fundamental frequency of the *short* call: *f*0 decreases with size. Dots represent averaged *f*0 values for each individual, and the yellow line the best fit of the linear model.

Mean *f*0 of the *short* call decreased with size (figure 3 and table 1). There was no size effect on the mean *f*0 of the *classic* call (table 1) or on the formant dispersion (table 1).

**Table 1.** Model results: linear mixed effect models testing the effect of size (total head length, ‘THL') on the source (mean *f*0) and filter (formant dispersion) parameters of the *short* and *classic* calls. Significance indicated with asterisks.

		predictors		scaled residuals					*p*-value	interpretation
		intercept	THL	min.	1Q	median	3Q	max.		
mean *f*0: short call	estimates	2064.01	−25.74	−2.78	−0.60	−0.01	0.49	3.73	0.041*	decrease with size
	s.e.	645.95	12.16							
	*t*-value	3.195	−2.117							
mean *f*0: classic call	estimates	1255.38	−5.95	−2.51	−0.46	0.02	0.52	2.63	0.686	no effect
	s.e.	676.01	12.66							
	*t*-value	1.857	−0.470							
formant dispersion	estimates	407.87	9.07	−1.86	−0.82	−0.03	0.76	2.42	1	no effect
	s.e.	1175.28	21.98							
	*t*-value	0.35	0.41							

### 3.2. Sex

Sex had no effect on the acoustic parameters of either call type (pDFA: *p* ≥ 0.3 in both cases; table 2), nor on the sound duration investigated separately (*p* > 0.1 in both cases; table 3).

**Table 2.** Results of the permuted discriminant function analysis for the *short* and *classic* call types, using 16 acoustic parameters in reduced dimensions. Significance indicated in italics.

result	short call	classic call
no. sex categories (levels of test factor)	2	2
no. individuals	26	24
total no. calls	574	159
=no. calls selected	24	22
correctly classified (%)	69.04	73.36
chance level (%)	69.08	72.10
*p-*value for classified	*0.51*	*0.37*
correctly cross-classified (%)	55.41	61.84
chance level for cross-classified (%)	55.60	55.90
relative cross-classification level	1.00	1.11
*p*-value for cross-classified	*0.51*	*0.26*
interpretation	*no effect*	*no effect*

**Table 3.** Model results: linear mixed effect models testing the effect of sex on sound duration of the *short* and *classic* calls.

		predictors		scaled residuals					*p*-value	interpretation
		intercept	sex	min.	1Q	median	3Q	max.		
sound duration (s): short call	estimates	0.22	−0.04	**−**3.32	−0.44	−0.07	0.38	7.93	>0.5	no effect
	s.e.	0.99	0.02							
	*t*-value	0.22	−0.71							
sound duration (s): classic call	estimates	−6.06	−0.33	−1.78	−0.47	−0.13	0.33	6.29	>0.1	no effect
	s.e.	5.23	0.23							
	*t*-value	−1.18	−1.42							

### 3.3. Partner similarity

**Figure 4.**
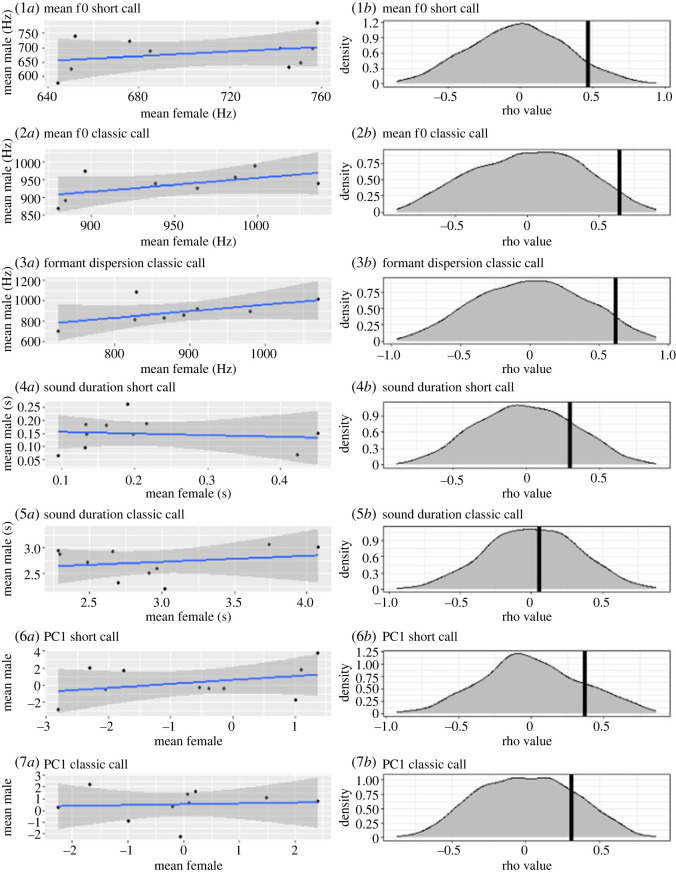
Observed relationship strength (*a*) and significance (*b*) of vocal similarity between partners, comparing mean *f*0 of the *short call* (1), mean *f*0 of the *classic call* (2), formant dispersion of the *classic call* (3), mean sound duration of the *short call* (4), mean sound duration of the *classic call* (5), scores of the 1st PC of the *short call* (6), and scores of the 1st PC of the *classic call* (7). While none of these parameters show a significant effect of partnership after Bonferroni adjustment, there is a clear tendency in the source and filter parameters of both call types to match between partners.

The mean *f*0 of short and classic call, as well as formant dispersion of the *classic* call tended to be more similar between partners (table 4 and figure 4), although the relationship remained statistically insignificant. Mean durations and scores of the first PC of both call types were not more similar between partners than between randomly assigned birds (table 4 and figure 4).

**Table 4.** Model results: correlation analysis testing similarity of different acoustic parameters between partners versus random birds.

parameter	*p*-value	*r*-value	interpretation
mean *f*0: short call	0.072	0.47	not significant; tendency to match
mean *f*0: classic call	0.036	0.64	not significant; tendency to match
formant dispersion: classic call	0.050	0.62	not significant; tendency to match
mean duration: short call	0.193	0.30	no effect
mean duration: classic call	0.444	0.06	no effect
PC1: short call	0.163	0.37	no effect
PC1: classic call	0.194	0.31	no effect

## 4. Discussion

We investigated the influence of size and sex on the acoustic parameters of little auk social calls, and considered partner similarity in the acoustic traits. Size had an effect on the source-related parameter (mean fundamental frequency) of one of the call types, the *short call*, with an increase in *f*0 with caller size (head length), but not on the other tested parameters. We found no influence of sex on either of the common call types. While we found no strong evidence on partner vocal similarity, there seemed to be a tendency for a matched *f*0 and formant dispersion between partners, particularly in the *classic call*.

### 4.1. Fundamental frequency and body size

Mean fundamental frequency is a common and reliable indicator of body size across taxa [44]. Here, we found that adult body size was reflected in the *f*0 of their *short* calls—that is, larger individuals produced calls of lower fundamental frequencies. This also seems to be the case in the little auk during ontogeny; as the chicks grow, the mean *f*0 of their calls becomes lower, reflecting changes in body size [32]. Although seabirds remain quite understudied in this respect, the same negative relationship between *f*0 and body size has been observed in the African penguins [5]. Other fundamental frequency parameters were shown to correlate with the overall body condition of great frigatebirds (*Fregata minor*) [50], and crested auklets (*Aethia cristatella*) [4]. While it is unclear whether the little auks perceive this difference in vocalizations, it is possible that fundamental frequency parameters may serve as indicators of the individual's overall health, as reflected by body size or motor control of the syrinx [51]—however, dedicated studies would be necessary to understand whether this is in fact the case.

Interestingly, there was no influence of size on the *f*0 of the *classic call.* Little auk call types vary greatly [31], and likely serve very different functions. The *classic call* is a long, multi-syllable vocalization uttered in a variety of contexts, including by birds sitting inside their nest chambers, escaping predators, flying over the colony alone or in a group. For species that depend on individual recognition to maintain crucial long-term partnerships, life in dense colonies may require extreme adjustments to signal identity [52–54]. In a social situation as complex as the little auk colony, such an elaborated vocalization may serve as an indicator of identity, maybe at the expense of other information, such as cues to size.

While *f*0 is mainly determined by the length of the larynx in mammals [44], avian syrinx is a much more complex structure, shown to allow for production of size-independent, or even multiple *f*0 within one vocalization [51]. Our results suggest that seabirds, or at least the little auk, are capable of both conveying honest cues to size (*short call*) and size-independent vocal modulations (*classic call*).

### 4.2. Formants and body size

Because the filtering process in mammals is strictly defined by the anatomical length of their vocal tract, formants are often very good indicators of body size in this group [44]. However, this relationship is neither obvious nor universal across the animal kingdom—particularly in birds, whose vocal production system is both more complex than that of mammals [51] and lacking the strict anatomical constraints by surrounding structures. Some species show modifications that distort the acoustic signal, such as tracheal prolongation [7]. As a result, the sender can not only ‘sound larger', which is beneficial in species with a preference for larger mates, but also produce signals of lower frequencies and an amplified output, that would propagate better through the environment [9], improving their long-distance communication.

Here, we found no indication of body size in the formant frequencies of little auk *classic* *calls*. This is line with previous research on birds, where formant frequencies were shown not to [5,54] or only weakly [10] indicate body size. Unlike the *short call*, the *classic call* is produced with an extended neck (either in flight, or posturing while seated), which might suggest active modification of the output sound. Interestingly, the *classic* call of little auks is often used in situations that might require long-distance transmission: for example, signalling from within a nest chamber [31]. This might imply that this call type is fine-tuned for effective communication at a distance.

While formant frequencies might not be a honest cue to size in birds, they should nevertheless depend on, and hence reflect, the total length of the vocal tract. Here, we were only able to measure the head length, as the distance between the back of the skull and the tip of the beak, which is just a part of the filter and does not reflect the overall vocal tract length. While THL [33] and beak length and/or width [5,50] were used as body size proxies in birds in similar studies, we do recognize that this is still not a standard measure, and it might render cross-species comparisons complicated. Since we studied living birds in a no-kill set-up, it was not possible to measure the total length and structure of the vocal tract of each focal individual. Further investigations into the topic might be interesting, should carcasses of naturally deceased birds become available, allowing full measurements and experiments with artificial air-flow through the excised vocal tract [55].

### 4.3. Sex differences

We did not find any evidence for encoding of information about the sex of the caller in the acoustic structure (defined by the 16 acoustic parameters we extracted) in two common calls of little auk calls. The negligible sexual dimorphism in this species [36] could explain the lack of information about sex in parameters that often reflect body size (e.g. fundamental frequency measures or formant dispersion). In addition, spectral properties of seabird calls do not seem to commonly indicate sex (however, see the yelkouan shearwaters (*Puffinus yelkouan*) with extremely reliable vocal differences between sexes [27]). Even species that do show sexual dimorphism in vocal tract anatomy might not encode sex in their vocalizations (as in e.g. herring gull, *Larus argentatus* [54]). However, we could have expected the temporal properties of the calls to differ between the sexes. Here, we specifically looked at the duration of little auk calls in relation to sex—still, there was no effect. In other species, some information on the caller's sex can also be conveyed by the temporal patterns of their vocalizations. For example, king penguins (*Aptenodytes patagonicus*) show a sex-specific syllable arrangement [2]. In the Cape gannet (*Morus capensis*), vocal cues to sex are encoded in the temporal rates of call displays [3]. While it seems unlikely that this is the case with little auk—*classic call* is produced as a single utterance, and the *short call* as a single vocalization or part of a bout during vocal exchanges with neighbours—no information about calling rates is currently available for the species, and the question remains to be tested. We suggest that other means of sex recognition, such as olfactory cues [56], should be considered in future experiments.

### 4.4. Partner similarity

Little auks mate assortatively regarding various morphological and physiological traits [17,36]. We thus expected to find significant similarities between partners' vocalizations, at least for parameters that were expected to be related to body size. This was not the case for any of the tested parameters of either call types. Since we have also found little effect of body size on the vocal output, the absence of partner similarity could be due to the absence of size encoding in given aspects of adult vocalizations. However, while we showed no statistically significant patterns, there seems to be a tendency for little auk partners to match in their mean *f*0 and formant dispersion (figure 4, 1–3). Little auk partners are known to match in their physiological profiles [17], namely differences between baseline and stress-induced corticosterone levels. Since vocal output can be influenced by hormones [26], it is possible that the apparent vocal similarity between little auk partners reflects physiological rather than morphological similarities.

Aside from being a result of morphological or physiological similarities, vocal similarity can be a result of vocal learning or social exposure. In some avian species, partners match their calls through a phenomenon termed ‘vocal convergence'. For example, raven (*Corvus corax*) partners use similar long-distance calls to improve communication at a distance [57], but otherwise are not vocally akin. Interestingly enough, in the little auk the tendency to match was stronger in the *classic call*, which we believe is used in long-distance communication, than in the short-range *short call.* Little auks share their parenting efforts equally and coordinate their foraging trips [30,58], which likely requires behavioural adjustments between the partners. A long distance call that is easily recognizable between partners could play a role in such coordination. On the other hand, calls of the African penguins (*Spheniscus demersus*) come to be more acoustically similar to their partner's and neighbours' as the animals become more familiar [59]. It is thus possible that species maintaining long-term partnerships will show vocal convergence between partners—and this might be the case of the little auks.

Because the sample size for this analysis was rather small—we were only able to record *classic* calls of both partners in eight nests, and *short* calls in nine nests—further analyses with a larger sample size should be performed to verify these findings. Such data on seabird partners' vocalizations are very rare and challenging to acquire, making even exploratory investigations noteworthy. However, once more data become available, this question should be revisited with more statistical power. In particular, access to animals of known relationship history (i.e. newly mated birds versus long-term partners) would help disentangle the potential physiological and social influences on their vocal output. While such data could be challenging to obtain from free ranging seabirds, experiments in controlled conditions or data collection from more easily accessible models would prove very useful. This could further help us understand whether the matching of certain traits is a result or driver of partnership in different assortatively mating groups [22].

## 5. Conclusion

Overall, we found that the fundamental frequency of little auk *short* calls carries information on body size. However, there seems to be no cues to sex in little auk vocalizations. While we found no strong vocal similarity between the partners, there seems to be a tendency to match source and filter parameters—yet more data would be necessary to fully investigate this question. While we do not understand yet how little auks come to form their partnerships, this study indicates that factors other than vocal cues are likely at play.

## Data Availability

Raw data generated in this study are available at https://osf.io/wp2uk/?view_only=feb0554f579c4cc08f6accc3e81af200. The data are provided in electronic supplementary material [61].
